# Benzophenanthridine Alkaloid Chelerythrine Elicits Necroptosis of Gastric Cancer Cells via Selective Conjugation at the Redox Hyperreactive C-Terminal Sec^498^ Residue of Cytosolic Selenoprotein Thioredoxin Reductase

**DOI:** 10.3390/molecules28196842

**Published:** 2023-09-28

**Authors:** Minghui Liu, Shibo Sun, Yao Meng, Ling Wang, Haowen Liu, Wuyang Shi, Qiuyu Zhang, Weiping Xu, Bingbing Sun, Jianqiang Xu

**Affiliations:** 1 School of Life and Pharmaceutical Sciences (LPS), Panjin Institute of Industrial Technology (PIIT), Dalian University of Technology, Panjin 124221, China; 2 School of Ocean Science and Technology (OST), Key Laboratory of Industrial Ecology and Environmental Engineering (Ministry of Education), Dalian University of Technology, Panjin 124221, China; 3 State Key Laboratory of Fine Chemicals, School of Chemical Engineering (CE), Dalian University of Technology, Dalian 116023, China

**Keywords:** thioredoxin reductase, chelerythrine, sanguinarine, necroptosis, gastric cancer, selenocysteine

## Abstract

Targeting thioredoxin reductase (TXNRD) with low-weight molecules is emerging as a high-efficacy anti-cancer strategy in chemotherapy. Sanguinarine has been reported to inhibit the activity of TXNRD1, indicating that benzophenanthridine alkaloid is a fascinating chemical entity in the field of TXNRD1 inhibitors. In this study, the inhibition of three benzophenanthridine alkaloids, including chelerythrine, sanguinarine, and nitidine, on recombinant TXNRD1 was investigated, and their anti-cancer mechanisms were revealed using three gastric cancer cell lines. Chelerythrine and sanguinarine are more potent inhibitors of TXNRD1 than nitidine, and the inhibitory effects take place in a dose- and time-dependent manner. Site-directed mutagenesis of TXNRD1 and in vitro inhibition analysis proved that chelerythrine or sanguinarine is primarily bound to the Sec^498^ residue of the enzyme, but the neighboring Cys^497^ and remaining N-terminal redox-active cysteines could also be modified after the conjugation of Sec^498^. With high similarity to sanguinarine, chelerythrine exhibited cytotoxic effects on multiple gastric cancer cell lines and suppressed the proliferation of tumor spheroids derived from NCI-N87 cells. Chelerythrine elevated cellular levels of reactive oxygen species (ROS) and induced endoplasmic reticulum (ER) stress. Moreover, the ROS induced by chelerythrine could be completely suppressed by the addition of *N*-acetyl-L-cysteine (NAC), and the same is true for sanguinarine. Notably, Nec-1, an RIPK1 inhibitor, rescued the chelerythrine-induced rapid cell death, indicating that chelerythrine triggers necroptosis in gastric cancer cells. Taken together, this study demonstrates that chelerythrine is a novel inhibitor of TXNRD1 by targeting Sec^498^ and possessing high anti-tumor properties on multiple gastric cancer cell lines by eliciting necroptosis.

## 1. Introduction

Cytosolic selenoenzyme thioredoxin reductase 1 (TXNRD1) plays important roles in various human diseases and is a vital target in cancer therapy [1,2,3]. TXNRD1 regulates cellular redox homeostasis and supports cell proliferation and survival when encountering unwanted oxidative damage from cell metabolism and external stress [4,5,6]. In addition to TXNRD, or the entire thioredoxin (TXN) system, glutathione (GSH) is the most abundant antioxidant in cellular components [7,8]. It works cooperatively with the TXN system to establish a cellular antioxidant defense line, along with antioxidant enzymes such as glutathione peroxidases (GPXs), peroxiredoxins (PRXs), and so on [9,10].

Cancer cells up-regulate the expression of TXNRD1 to control excessive reactive oxygen species (ROS) generated from anabolic and catabolic processes [11,12]. This makes TXNRD1 an appealing target for cancer therapy by disturbing cell redox balance [13,14]. Numerous TXNRD1 inhibitors have been discovered in the last decades [15], including three FDA-approved drugs like auranofin [16], ebselen [17], and dimethyl fumarate (DMF) [18]. Additionally, two newly discovered high-efficacy molecules, TRi-1 and TRi-2, have also been identified [19]. Some natural compounds, such as parthenolide [20], piperlongumine [21], and shikonin [22], have also been found to inhibit TXNRD1.

Gastric cancer (GC) is one of the most common tumors and ranks as the third leading cause of new death worldwide [23]. The expression level of TXNRD1 is higher in GC tissues compared to adjacent normal tissues. Additionally, high expression of TXNRD1 is significantly associated with poor outcomes in patients with gastric cancer [24]. Recent studies have revealed that targeting TXNRD1 with small molecules is a viable approach to achieving therapeutic activity and selectivity, indicating the anti-cancer potential of inhibiting TXNRD1 in gastric cancer [25,26].

A recent report has shown that sanguinarine (San) is an inhibitor of TXNRD1, indicating that benzophenanthridine alkaloid is a novel chemical entity among the inhibitors of TXNRD1 [27]. Meanwhile, benzophenanthridine alkaloids have been reported to be potentially valuable due to their anti-tumor activities [28,29,30]. However, it is largely unclear whether the inhibitory activity of TXNRD1 commonly occurs in benzophenanthridine alkaloids that contain the quaternary nitrogen group.

**Figure 1:**
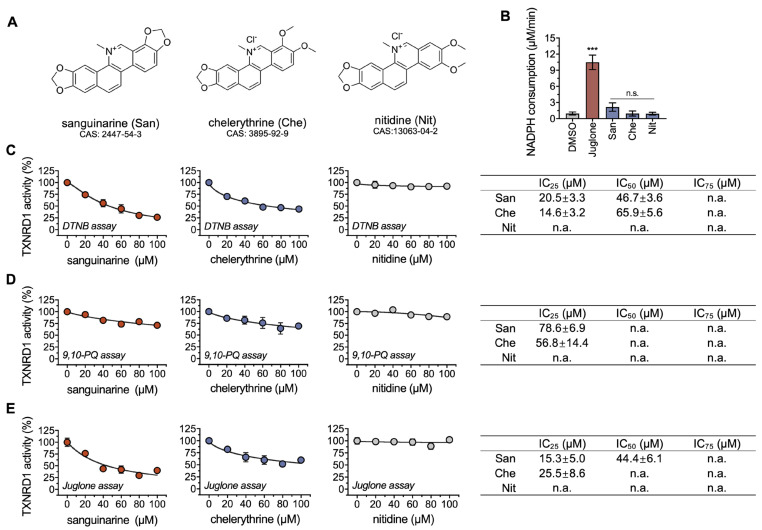
**Chelerythrine inhibits the recombinantly purified rat TXNRD1.** (**A**) Chemical structures of sanguinarine (San), chelerythrine (Che), and nitidine (Nit). (**B**) Reduction of indicated compounds by TXNRD1. The reaction mixture contained 30 nM TXNRD1, 200 μM NADPH, and 30 μM compounds. (**C**–**E**) Dose-response inhibition of TXNRD1 by chelerythrine and sanguinarine. TXNRD1 was incubated with the indicated compounds for 1 h at room temperature. Then, the activity of TXNRD1 was measured by three classical assays. The IC_50_ values, together with the IC_25_ and IC_75_ values, are shown as mean ± SEM in the table panel on the right. The values of IC_25/50/75_ were estimated from the dose-response curves by nonlinear curve fitting using Prism 8. n.a. means not applicable, and n.s. represents not significant, ***, *p* < 0.001.

In addition to sanguinarine, chelerythrine (Che) and nitidine (Nit) are two benzophenanthridine alkaloids extracted from the roots of the medicinal plants *Chelidonium majus* L. (*C. majus*) [31] and *Zanthoxylum nitidum (Roxb.) DC. (ZN)* [32], respectively, with a broad spectrum of anti-cancer activity against various types of cancer cells [33]. Differing from sanguinarine, chelerythrine and nitidine contain ortho-methoxy groups at the A ring (Figure 1A), which may alter their pharmacological activity. Till now, the cytotoxicity profiles and anti-tumor mechanisms of benzophenanthridine alkaloids such as sanguinarine, chelerythrine, and nitidine in gastric cancers have been vague. Hence, the interaction between TXNRD1 and Che or Nit is worth disclosing.

In the present study, we focused on the targeting and inhibitory effects of benzophenanthridine alkaloids, commenced on sanguinarine, and further investigated two more compounds, including chelerythrine and nitidine, on their inhibitory effects on TXNRD1 in detail using recombinantly purified wild-type enzymes and their different mutant variants. Our data clearly revealed that chelerythrine and sanguinarine irreversibly inhibit the activity of TXNRD1 while specifically targeting Sec^498^ of the enzyme, presenting significant anti-cancer activities through necroptotic cell death. So far, our study provides new insights into the discovery of benzophenanthridine alkaloids as novel TXNRD1 inhibitors and discloses their cytotoxicity mechanism.

## 2. Results

### 2.1. Chelerythrine and Sanguinarine Are Inhibitors of TXNRD1

TXNRD1 is a redox-active reductase that catalyzes multiple low-weight substrates, such as quinones, disulfide compounds, and selenides [34]. NADPH is the electron donor for TXNRD1 to reduce its substrates. The oxidation activity of NADPH can be monitored to verify whether an electron transfer occurs between TXNRD1 and the aforementioned three benzophenanthridine alkaloids. Compared with DMSO, no significant change was observed in these three compounds. In contrast, juglone, a substrate of TXNRD1, exhibited strong NADPH oxidation (Figure 1A,B). Clearly, these three compounds, in particular chelerythrine and nitidine, are not substrates of TXNRD1. It should be noted that we observed a substantial decrease in absorbance at 340 nm (from ~1.2 to ~1.0) at the first minute of the reaction in the absence of TXNRD1 and NADPH. We suspect that the phenomenon was caused by the compounds themselves and that it did not affect the enzyme-based activity test.

We then assessed the inhibitory effects of three benzophenanthridine alkaloids on wild-type TXNRD1. After a 1 h incubation, chelerythrine and sanguinarine, rather than nitidine, exhibited dose-response inhibitions on TXNRD1 in the DTNB reduction (Figure 1C), 9,10-PQ reduction (Figure 1D), and juglone reduction (Figure 1E). As known, DTNB and 9,10-PQ are Sec-dependent substrates of TXNRD1, while juglone is not. In the DTNB-reduction activity assay, the IC_50_ value of chelerythrine was 65.9 μM, about 1.5-fold higher than that of sanguinarine (46.7 μM), suggesting that sanguinarine is more potent. Additionally, the inhibitory effects of sanguinarine on TXNRD1’s activity were consistent with a previous study [27]. These results indicated that chelerythrine is a novel inhibitor of TXNRD1 in a dose-dependent manner and led us to further investigate the inhibition details of chelerythrine and sanguinarine on TXNRD1.

### 2.2. Chelerythrine and Sanguinarine Primarily Target the Hyperreactive Selenocysteine Residues

**Figure 2:**
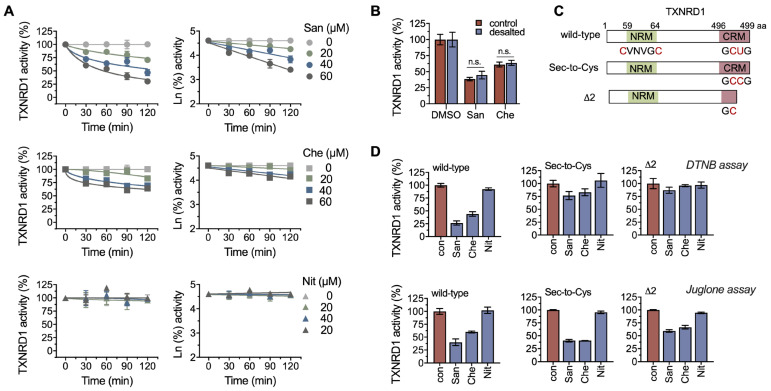
**Chelerythrine and sanguinarine irreversibly inhibit TXNRD1 through selenol/thiol modifications.** (**A**) Time-course inhibition of TXNRD1 by chelerythrine and sanguinarine. TXNRD1 was incubated with three compounds separately, and afterwards, the enzyme activity was assayed at various indicated times. (**B**) Irreversible inhibition of TXNRD1 by chelerythrine. TXNRD1 was incubated with 100 μM sanguinarine or chelerythrine for 1 h. Then the samples were subjected to NAP-5^TM^ desalting columns to remove the free compounds. The activity of the eluted fractions was determined by using DTNB as the substrate. (**C**) Schematic diagram of TXNRD1 and its Sec-to-Cys mutant and UGA-truncation delta2 form used in this study. (**D**) Priority modification of selenocysteine by sanguinarine or chelerythrine. TXNRD1 and its mutants were incubated with 100 μM sanguinarine or chelerythrine for 1 h. The relative enzyme activity was determined using both the DTNB assay and the juglone assay. n.s. means not significant.

Previously, it was reported that benzophenanthridine alkaloids can form adducts with the thiol groups of proteins [35,36]. In this experiment, we tested the possible modification of chelerythrine and sanguinarine on TXNRD1. The results showed that chelerythrine and sanguinarine significantly inhibited TXNRD1 in a time-dependent manner (Figure 2A), with a *k*_inact_ of 6.29 × 10^5^ μM^−1^ min^−1^ for chelerythrine and 1.56 × 10^−4^ μM^−1^ min^−1^ for sanguinarine. Removing the free compounds using a desalting column could not rescue the enzyme activity, indicating that chelerythrine and sanguinarine irreversibly inhibit TXNRD1 (Figure 2B).

TXNRD1 is a selenoprotein that typically functions as a dimer. The catalytic pocket contains an N-terminal GSR-like “CVNVGC” motif from one subunit and a C-terminal selenocysteine-containing “GCUG” redox motif from the other subunit (Figure 2C) [37,38,39]. Here, we used two TXNRD1 mutants, the Sec-to-Cys and Δ2 mutants of the enzyme, to determine the residue(s) that may be targeted by chelerythrine. The Sec-to-Cys mutant indicated that the selenocysteine at position 498 was replaced with cysteine. The Δ2 mutant indicated that the last two residues (selenocysteine and glycine) of TXNRD1 were deleted. Both chelerythrine and sanguinarine exhibited strong inhibition on wild-type TXNRD1, weaker inhibition on its Sec-to-Cys mutant, and no inhibition on the Δ2 mutant. These above-mentioned results suggest that chelerythrine and sanguinarine may inhibit TXNRD1 by modifying the Sec^498^ residue of the enzyme.

However, the possibility that chelerythrine and sanguinarine also modify the Cys residues of TXNRD1 (Figure 2D) cannot be ruled out. Juglone has been reported as a Sec-independent substrate of TXNRD1, and the reduction of juglone by TXNRD1 is mainly dependent on the FAD/CVNVGC motif [40,41]. The inhibition of chelerythrine and sanguinarine on juglone reduction suggests that the cysteine residue(s) of TXNRD1 can be targeted by chelerythrine or other benzophenanthridine alkaloids (Figure 2D).

### 2.3. Chelerythrine and Sanguinarine Suppress the Proliferation of Gastric Cancer Cells

**Figure 3:**
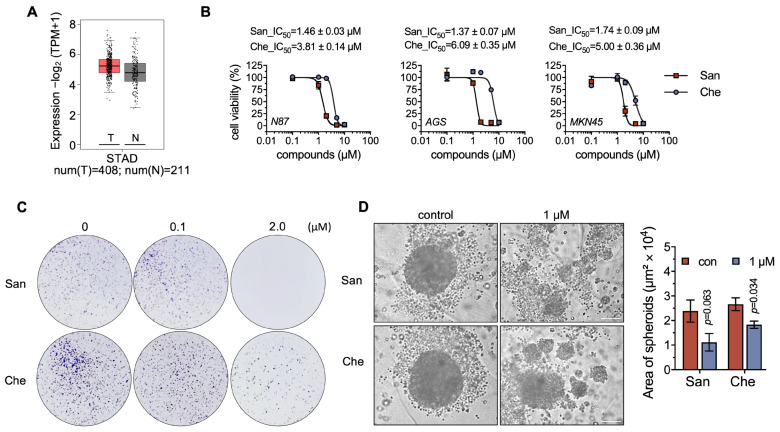
**Chelerythrine and sanguinarine can suppress the proliferation of NCI-N87 cells.** (**A**) Expression level of TXNRD1 in gastric cancer. The analysis was performed using GEPIA2 (http://gepia2.cancer-pku.cn (accessed on 1 July 2023)). The red box indicates the tumor group, and the gray box represents the normal group. (**B**) Cytotoxicity of chelerythrine (Che) and sanguinarine (San) on gastric cancer cells. NCI-N87, AGS, and MKN45 cells were treated separately with chelerythrine and sanguinarine for 24 h. Afterwards, MTT assay was used to measure the cell viability. IC_50_ values are shown as mean ± SEM. (**C**) Suppression of NCI-N87 cell proliferation upon treatment with chelerythrine or sanguinarine. NCI-N87 cells were incubated with sanguinarine or chelerythrine for 12 h. After incubation, the cells were cultured for an additional two weeks before microscopic observation. (**D**) Tumor spheroid formation assay of NCI-N87 cells being treated with either sanguinarine or chelerythrine. Multicellular tumor spheroids were grown from NCI-N87 cells for 1 day and then treated with chelerythrine and sanguinarine for 3 days. Spheroids were documented under a microscope, scale bar, 100 μm.

TXNRD1 is commonly overexpressed in cancer cells [42]. We analyzed the expression level of TXNRD1 in gastric cancer using the GEPIA2 software (http://gepia2.cancer-pku.cn (accessed on 1 July 2023)). Compared to normal tissues, TXNRD1 was up-regulated in gastric tumors, suggesting that TXNRD1 could be a potential cellular target for the treatment of gastric cancers (Figure 3A). To elucidate the anti-tumor mechanism of chelerythrine and sanguinarine, we then assessed the cytotoxicity of chelerythrine and sanguinarine in gastric cancer cell lines. After being treated with the indicated compounds for 24 h, the cell viability of NCI-N87 cells decreased (Figure 3B). The IC_50_ values for chelerythrine and sanguinarine in NCI-N87 cells were 3.81 μM and 1.46 μM, respectively.

In addition to NCI-N87 cells, we tested the cytotoxicity of chelerythrine and sanguinarine on MKN45 and AGS cells (Figure 3B). The cytotoxicity profiles of chelerythrine and sanguinarine were highly similar in these three cancer cells, indicating that benzophenanthridine alkaloids, such as chelerythrine and sanguinarine, may exhibit anti-tumor activity in multiple gastric cancer cell lines. Additionally, the colony formation assay demonstrated that 2 μM chelerythrine or sanguinarine suppressed cell proliferation activity in NCI-N87 cells (Figure 3C).

Tumor spheroids can mimic important aspects of solid tumors, such as hypoxia and metabolic heterogeneity. In this study, we developed tumor spheroids using NCI-N87 cells and subsequently treated the spheroids with chelerythrine and sanguinarine. The size of the spheroids indicated that the growth of tumor spheroids was inhibited by 1 μM chelerythrine to about 70% and by 1 μM sanguinarine to approx. 50% (Figure 3D). A concentration of 5 μM chelerythrine and sanguinarine was also used in this assay, but it resulted in the rapid and forced cell death of the spheroids. These results proved that either chelerythrine or sanguinarine can suppress the proliferation of gastric cancer cells.

### 2.4. Chelerythrine and Sanguinarine Induce Oxidative Stress in Gastric Cancer Cells

**Figure 4:**
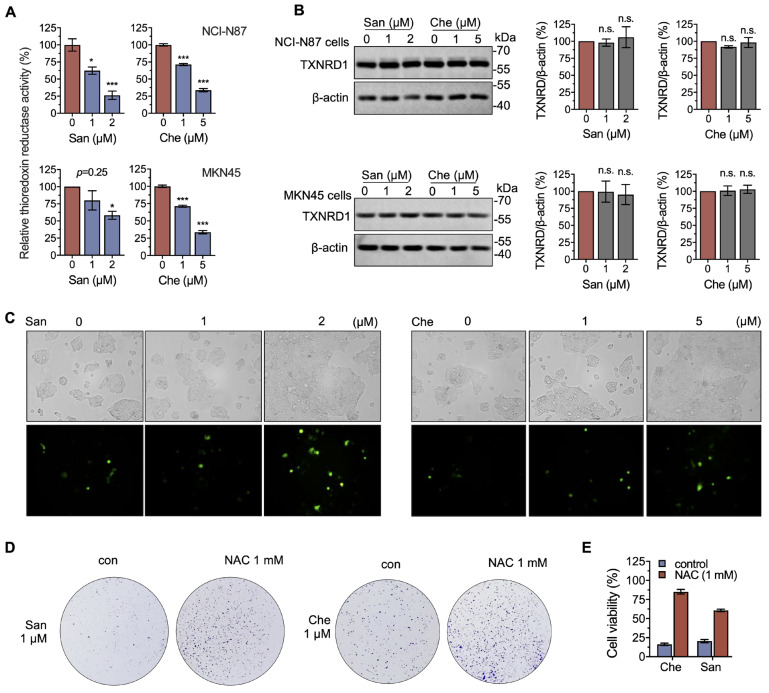
**Chelerythrine and sanguinarine induce oxidative stress by inhibiting cellular TXNRD activity.** (**A**) Both chelerythrine (Che) and sanguinarine (San) inhibit the thioredoxin reductase activity of cellular TXNRD1. NCI-N87 cells (upper panel) and MKN45 cells (lower panel) were treated with either chelerythrine or sanguinarine for 4 h, and the cellular TXNRD activity in cell lysates was determined by using an end-point insulin-coupled TXN assay. (**B**) Expression levels of TXNRD1 following treatment with chelerythrine or sanguinarine. TXNRD1 expression levels were determined after chelerythrine or sanguinarine treatment for 4 h (NCI-N87 cells (upper panel) and MKN45 cells (lower panel)). (**C**) Chelerythrine and sanguinarine increase cellular ROS levels. NCI-N87 cells were treated with chelerythrine or sanguinarine for 4 h. The level of cellular ROS was quantified by H_2_DCFH-DA and documented by fluorescence microscopy. (**D**) The antioxidant NAC rescues the proliferation of NCI-N87 cells from chelerythrine and sanguinarine. A total of 1 mM NAC was co-treated with 1 μM chelerythrine or sanguinarine for 12 h to measure the colony formation. (**E**) NAC counteracts the cytotoxicity of chelerythrine and sanguinarine. Cells were treated with 2 μM sanguinarine and 5 μM chelerythrine, with or without 1 mM NAC, for 24 h. n.s. means not significant, *, *p* < 0.05, ***, *p* < 0.001.

The cellular redox homeostasis is maintained by the TXN system and the GSH system. These two systems work cooperatively and competitively [5,43]. We next revealed whether the cellular redox balance was perturbed when treating gastric cells with chelerythrine and sanguinarine. We demonstrated that after being treated with either 5 μM chelerythrine or 2 μM sanguinarine for 4 h, cellular TXNRD1 activity in NCI-N87 cells was significantly reduced to approx. 25%, compared to the untreated control (Figure 4A). Around 50% of cellular TXNRD activity in MKN45 cells could be inhibited by 5 μM chelerythrine or 2 μM sanguinarine (Figure 4A). Notably, the expression level of TXNRD1 in these cells was not changed (Figure 4B), indicating that chelerythrine or sanguinarine decreased cellular TXNRD1 activity by directly inhibiting the enzyme.

Furthermore, by using the ROS probe DCFH-DA, we showed a dramatic increase in cellular ROS when treated with 5 μM chelerythrine or 2 μM sanguinarine (Figure 4C), indicating that chelerythrine and sanguinarine induce oxidative stress in NCI-N87 cells. Meanwhile, the anti-proliferation activity of chelerythrine and sanguinarine could be completely rescued by 1 mM NAC (Figure 4D), and the cytotoxicity of these two compounds was counteracted by NAC (Figure 4E). These results suggest that chelerythrine and sanguinarine induce cell death in a ROS-dependent manner.

### 2.5. Chelerythrine and Sanguinarine Promote Necroptosis in Gastric Cancer Cells

**Figure 5:**
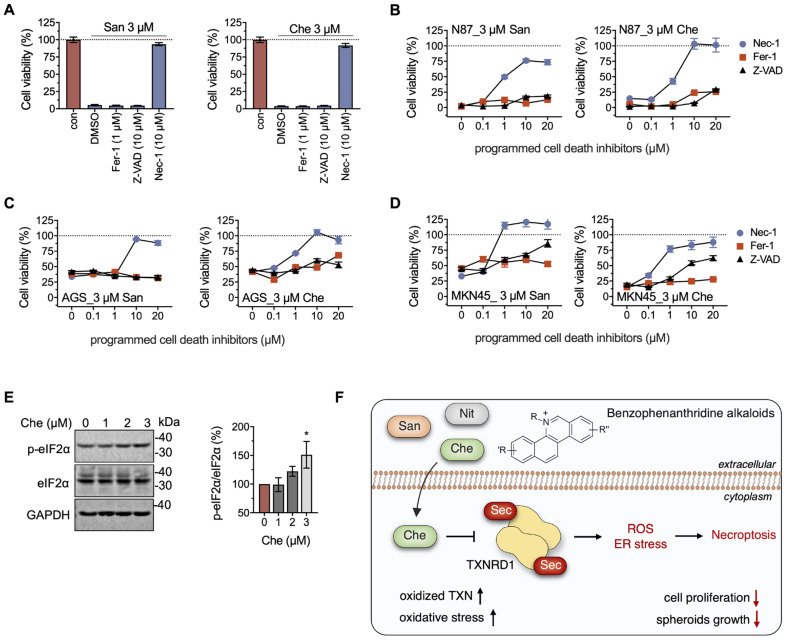
**Chelerythrine and sanguinarine elicit necroptosis in NCI-N87 cells by inducing ER stress.** (**A**) RIPK1 inhibitor Nec-1 suppresses cell death induced by chelerythrine (Che) or sanguinarine (San) in NCI-N87 cells. A total of 1 μM Fer-1, 10 μM Z-VAD, and 10 μM Nec-1 were used in this experiment. (**B**–**D**) Chelerythrine induces necroptosis in gastric cancer cells. Programed cell death inhibitors ranging from 0 to 20 μM were separately added to the cultured cells that were incubated with 3 μM of either chelerythrine or sanguinarine. After cell cultivation for 24 h, cell viability was determined using MTT assay. (**E**) Chelerythrine-induced ER stress in NCI-N87 cells. NCI-N87 cells were treated with the indicated concentrations of chelerythrine for 36 h, and the samples were analyzed using Western blots. (**F**) Schematic diagram of benzophenanthridine alkaloids like chelerythrine inducing necroptosis in gastric cancer cells. Herein, cellular consequences of chelerythrine may include up-regulation of oxidized TXN, increased oxidative stress, and suppression of cell proliferation, in particular hindering the spheroids’ growth. *, *p* < 0.05.

To further elucidate the anti-tumor mechanisms of chelerythrine and sanguinarine, especially stress-induced programmed cell death (PCD) in gastric cancer cell lines, we introduced PCD inhibitors to the incubation system. A total of 10 μM RIPK1 inhibitor Nec-1, instead of the ferroptosis inhibitor Fer-1 (1 μM) or the pan-caspase inhibitor Z-VAD-FMK (10 μM), rescued cell death induced by both chelerythrine and sanguinarine in NCI-N87 cells (Figure 5A). The protective effect of Nec-1 on chelerythrine and sanguinarine was observed at concentrations ranging from 0.1 μM to 1 μM. Furthermore, 10 μM Nec-1 completely rescued cell death induced by chelerythrine and sanguinarine (Figure 5B). Meanwhile, Nec-1 showed protective activity in both AGS and MKN45 cells, indicating that chelerythrine and sanguinarine induce necroptosis in multiple gastric cancer cell lines (Figure 5C,D).

Interestingly, Fer-1 and Z-VAD-FMK showed no effect on the rescue of cells against chelerythrine and sanguinarine at low concentrations and only slight amelioration at high concentrations. It can be inferred from the results that 3 μM chelerythrine promoted non-specific lipid peroxidation or DNA damage (Figure 5C,D). However, this circumstance did not ultimately lead to typical ferroptosis and apoptosis in gastric cancer cells [44,45,46].

Endoplasmic reticulum (ER) stress is one of the inducers of necroptosis [47,48,49]. Here, we found that the protein level of p-eIF2α, increased when the NCI-N87 cells were treated with 3 μM chelerythrine for 36 h, suggesting that unfolded protein response (UPR) is initiated by ER stress, which ultimately triggers necroptosis in NCI-N87 cells (Figure 5E,F).

## 3. Discussion

The high energetic needs for cell growth in cancer cells are naturally occurring but also unavoidably confer redox vulnerabilities in the chemotherapy of various cancers [50,51,52,53]. Antioxidant enzymes, such as TXNRD, glutathione reductase (GSR), and xCT/SLC7A11, are commonly up-regulated in cancer cells to counteract the unwanted oxidation derived from metabolism [51,54]. The perturbations of the cellular redox balance can trigger programmed cell death, including ferroptosis and apoptosis [45,50,55]. Here, we showed that two natural compounds, chelerythrine and sanguinarine, suppress the growth of gastric cancer cells by inhibiting TXNRD1 and inducing oxidative stress in NCI-N87 cells. TXNRD1 is a fundamental enzyme in regulating the reduced status of TXN and a further participant in protein oxidation repair, cystine reduction, and DNA synthesis [4,56,57]. Our data showed that after being exposed to either chelerythrine or sanguinarine for 4 h, cellular TXNRD1 activity rapidly decreased. The loss of activity in TXNRD1 led to ROS-dependent cell death, which supports a redox-targeting strategy in the chemotherapy of gastric cancer.

Apart from the stressed oligomerized forms [58,59], TXNRD1 is generally a homodimeric selenoenzyme with a highly reactive selenocysteine at position 498, which has been considered a potential drug target in cancer therapy [58,59,60]. We showed here that chelerythrine is capable of modifying the Sec^498^ residue of TXNRD1, which is consistent with most TXNRD1 inhibitors. Meanwhile, our previous results revealed that Cys^498^ could also be attacked by electrophiles [21,61,62,63,64]. This study also provides evidence that chelerythrine interacts with Cys^498^. It has been reported that inhibiting TXNRD1 with small molecules may convert the enzyme from antioxidant to pro-oxidant, a phenomenon known as SecTRAPs [42,65]. It can be inferred that the features of TXNRD1 also contribute to the anti-cancer properties of chelerythrine.

Chelerythrine, nitidine, and sanguinarine belong to the group of benzophenanthridine alkaloids. The anti-tumor activity of benzophenanthridine alkaloids has been revealed in recent years. Sanguinarine induces p53-dependent up-regulation of miR-16, leading to cell arrest and apoptosis, as well as inverting EMT through Wnt/β-catenin signaling [28,29,30]. Most studies have revealed that sanguinarine or chelerythrine promotes apoptosis in cancer cells through various mechanisms [33]. Surprisingly, we found that Nec-1, a RIPK1 inhibitor, could rescue the cytotoxicity of both chelerythrine and sanguinarine, indicating that these compounds induce necroptotic cell death in NCI-N87 cells. Meanwhile, it has been reported that chelerythrine promotes necroptosis signaling in gliomas [66]. These results suggest that the cytotoxicity of benzophenanthridine alkaloids in human cancers varies depending on cell type and genetic differences. However, we showed here that chelerythrine and sanguinarine induced necroptosis and ROS-dependent cell death, but the underlying mechanism has not been fully investigated, and this should be clarified in future studies.

Previous studies have shown that 3D cell culture is more representative of the *in vivo* environment than 2D culture [67]. In this study, we established a cultivation and assay for gastric tumor spheroids to overcome the limitations of 2D cell culture. The tumor spheroids exhibited the physiological characteristics of solid tumors, such as cell–cell contact formation, reduced proliferation, and hypoxia environment, all of which can impact the effectiveness of various anti-tumor drugs. Notably, the results showed that the growth of NCI-N87-derived spheroids was significantly inhibited, indicating the therapeutic potential of chelerythrine and sanguinarine in chemotherapy. Furthermore, the cellular effects of chelerythrine and sanguinarine were recently investigated in vivo [66,68,69]. The results suggest that chelerythrine, as well as sanguinarine, is a potential candidate for cancer chemotherapy and other diseases.

## 4. Materials and Methods

### 4.1. Chemicals and Reagents

The chemicals used in this study include sanguinarine (San, HY-N0052, MCE, Shanghai, China), chelerythrine chloride (Che, HY-12048, MCE), nitidine chloride (Nit, HY-N0498), z-VAD-FMK (Z-VAD, S81415, Yuanye, Shanghai, China), necrostatin-1 (Nec-1, HY-15760, MCE), nicotinamide adenine dinucleotide phosphate (NADPH, S10103, Yuanye), ferrostatin-1 (Fer-1, S81461, Yuanye), 5,5′-dithiobis-(2-nitrobenzoic acid) (DTNB, S19139, Yuanye), 5-Hydroxy-1,4-naphthoquinone (juglone, H47003, Sigma-Aldrich, St. Louis, MO, USA), 9,10-phenanthrene quinone (9,10-PQ, P106382, Aladdin, Shanghai, China), and *N*-acetyl-L-cysteine (NAC, A601127, Sangon, Shanghai, China).

The proteins used in this study include bovine insulin (S12033, Yuanye), recombinant rat TXNRD1 (16.8 U/mg), and recombinant human TXN1. The latter two proteins were recombinantly expressed in *E. coli* BL21(DE3) strains and chromatographically purified to homogeneity as one band on SDS gels, according to previously reported methods [59,70].

The antibodies used in this study include anti-TXNRD1 (1:5000, 67728, ProteinTech, Wuhan, China), anti-β-actin (1:20,000, 66009, Proteintech), anti-phospho-eIF2A (Ser51) (1:1000, R22946, Zenbio, Chengdu, China), anti-eIF2A (1:1000, 201137, Zenbio), anti-GAPDH (1:10,000, 60004-1-Ig, ProteinTech), goat anti-mouse IgG (SA00001-1, ProteinTech), and goat anti-rabbit IgG (SA00001-2, ProteinTech).

The critical commercial assays used in this study include the BCA protein kit (P0012, Beyotime, Shanghai, China) and Bradford protein kit (C503031, Sangon).

### 4.2. Cancer Cell Culture

Human gastric cancer cells NCI-N87 (CL-0169, Procell, Wuhan, China), AGS (CL-0022, Procell), and MKN45 (CL-0292, Procell) were cultured in RPMI 1640 medium (PM150110, Procell). All the cultured cells were supplemented with 10% fetal bovine serum (FBS, 164210, Procell), 100 U/mL penicillin, and 100 mg/mL streptomycin (P/S, C0222, Beyotime) in a humidified incubator (HealForce, Shanghai, China) with an atmosphere of 5% CO_2_ and a temperature of 37 °C.

### 4.3. Cell Viability

Cell viability was assayed using the MTT assay. Initially, cells were seeded at a density of 5000 cells per well in a 96-well plate and cultured overnight. The next day, cells were exposed to compounds for the indicated time. After incubation, the medium was removed, and a fresh medium containing 0.5 mg/mL of MTT was added and incubated continuously for 2–4 h. Subsequently, the formazan crystals were dissolved in 100 μL of dimethyl sulfoxide (DMSO), and the absorbance at 570 nm was measured with 630 nm as a reference by using a SpectraMax ABS plate reader (Molecular Devices, Sunnyvale, CA, USA).

### 4.4. Cell Proliferation

Cells were initially seeded at a density of 1000 cells per well in 6-well plates and allowed to adhere overnight. Cells were then exposed to sanguinarine or chelerythrine for 12 h. Subsequently, the cells were cultured for 1–2 weeks, with medium replaced every 3 days. After incubation, the colonies were fixed with a 4% paraformaldehyde solution for 1 h and stained with 0.1% crystal violet stain for 10 min.

### 4.5. Spheroid Formation

NCI-N87 cells (5000 cells per well) were seeded into ultra-low attachment 96-well plates with 4% Matrix gel (356234, Corning, Corning, NY, USA). The formed spheroids were then treated with compounds and grown for one week. Finally, experimental images of the spheroids were taken with a microscope (DSY-LV2000, Crisoptical, Beijing, China).

### 4.6. Western Blotting

Cancer cells (200,000 cells per well) were seeded into 6-well plates and treated with compounds for the indicated time. After incubation, cells were lysed using RIPA buffer containing protease inhibitors and phosphatase inhibitors. The protein concentrations were determined by BCA assay, and BSA was used as protein standard. In parallel, the cell lysate samples with equal amounts of proteins were separated using SDS-PAGE, and all protein bands were transferred into polyvinylidene difluoride (PVDF) membranes (0.45 μm, Millipore, Burlington, MA, USA). The membranes were blocked with 5% (*m*/*v*) non-fat milk for 60 min at room temperature and then blotted with primary antibodies overnight at 4 °C. After washing with TBST three times, the membranes were incubated with secondary antibodies at room temperature for 1 h and developed in ECL solution (AP34L024, Life-iLab, Shanghai, China).

### 4.7. Cellular TXNRD Activity

Cellular TXNRD activity was assayed by a TXN1-coupled end-point insulin assay [71]. In brief, an appropriate amount of cell lysates was added into a master mixture containing 80 mM Hepes buffer (pH 7.5), 15 μM TXN1, 300 μM insulin, 660 μM NADPH, and 3 mM EDTA. A reaction mixture without TXN1 was used as a background control. Samples were incubated at 37 °C for 30 min. Subsequently, 6.0 M guanidine hydrochloride (GuHCl), containing 1 mM DTNB and 20 mM EDTA (pH 7.5), was added to each well, and the whole microtitrate plate was shaken for 10 s. Subsequently, an end-point absorbance in each well at 412 nm was measured simultaneously, and the wells without enzymes were used as blanks. The TXNRD activities of cell lysates were normalized to protein concentration for accuracy comparison.

### 4.8. Inhibition of Compounds on Recombinant TXNRD1

Regarding the incubation system with compounds, TXNRD1 was first pre-reduced by 100 μM NADPH for 10 min and then treated with compounds at different concentrations as indicated in the experiments. After incubation, the TXNRD1 activity was determined by DTNB-reducing activity assay, unless otherwise noted. In the DTNB-reducing assay, the final reaction mixture contained 2.5 mM DTNB, 300 μM NADPH, and 10–20 nM wild-type TXNRD1 or its various mutants (100 nM) in 50 mM TE buffer (pH 7.5), and the enzyme activity was calculated by TNB^−^ formation at 412 nm (*ε*_TNB_^–^ = 13,600 M^−1^ cm^−1^).

In the 9,10-PQ- and juglone-reducing activity assays, the final mixture contained 30 nM wild-type TXNRD1 or its various mutants, 30 μM 9,10-PQ or juglone, and 200 μM NADPH in 50 mM TE buffer (pH 7.5). The enzymatic activity of 9,10-PQ or juglone reduction was calculated by NADPH oxidation at 340 nm (ε_NADPH_ = 6200 M^−1^ cm^−1^). All reactions were performed three times by using an Infinite 200 PRO plate reader (Tecan, Männedorf, Switzerland) at 25 °C. The same reaction mixture lacking the enzyme was used as a reference.

### 4.9. ROS Detection

NCI-N87 cells were seeded into 12-well plates at 100,000 cells per well and treated with different doses of sanguinarine and chelerythrine for 4 h. After incubation, the medium was replaced with serum-free culture medium containing 10 μM DCFH-DA and incubated for 30 min at 37 °C. Cells were washed three times with serum-free cell culture medium and then documented by fluorescence microscopy.

### 4.10. Statistical Analysis

All experiments were performed in triplicate, and the data were presented as the mean ± SEM. The IC_50_ values were calculated by non-linear curve fitting using Prism 8.0 (Graphpad, version 8.4.0). Statistical differences between the two groups were analyzed using the Student’s *t*-test. Comparisons among multiple groups were statistically assessed by one-way analysis of variance (ANOVA), followed by a post hoc Scheffe test. The significant differences between groups were defined as * *p* < 0.05, ** *p* < 0.01, *** *p* < 0.001; n.s. means not significant.

## 5. Conclusions

This study demonstrated that chelerythrine is a novel inhibitor of TXNRD1 and revealed that both chelerythrine and sanguinarine can inhibit TXNRD1’s activity by modifying the selenocysteine residue. The anti-gastric cancer activity of chelerythrine and sanguinarine was assessed to promote ROS-dependent necroptosis in NCI-N87 cells. The results obtained in this study would be useful in finding novel inhibitors of TXNRD1 and provide new insights into the anti-tumor activity of benzophenanthridine alkaloids.

## Data Availability

Not applicable.
